# Searching the Cytochrome P450 Enzymes for the Metabolism of Meranzin Hydrate: A Prospective Antidepressant Originating from Chaihu-Shugan-San

**DOI:** 10.1371/journal.pone.0113819

**Published:** 2014-11-26

**Authors:** Xi Huang, Ying Guo, Wei-hua Huang, Wei Zhang, Zhi-rong Tan, Jing-bo Peng, Yi-cheng Wang, Dong-li Hu, Dong-sheng Ouyang, Jian Xiao, Yang Wang, Min Luo, Yao Chen

**Affiliations:** 1 Department of Clinical Pharmacology, Xiangya Hospital, Central South University, Changsha, Hunan 410078, China; 2 Institute of Clinical Pharmacology, Central South University, Hunan Key Laboratory of Pharmacogenetics, 110 Xiangya road, Changsha, Hunan 410078, China; 3 Laboratory of Ethnopharmacology, Institute of Integrated Traditional Chinese and Western Medicine, Xiangya Hospital, Central South University, 87 Xiangya Road, 410008 Changsha, China; Universidade Federal do Rio de Janeiro, Brazil

## Abstract

Meranzin hydrate (MH), an absorbed bioactive compound from the Traditional Chinese Medicine (TCM) Chaihu-Shugan-San (CSS), was first isolated in our laboratory and was found to possess anti-depression activity. However, the role of cytochrome P450s (CYPs) in the metabolism of MH was unclear. In this study, we screened the CYPs for the metabolism of MH *in vitro* by human liver microsomes (HLMs) or human recombinant CYPs. MH inhibited the enzyme activities of CYP1A2 and CYP2C19 in a concentration-dependent manner in the HLMs. The *K_m_* and *V_max_* values of MH were 10.3±1.3 µM and 99.1±3.3 nmol/mg protein/min, respectively, for the HLMs; 8.0±1.6 µM and 112.4±5.7 nmol/nmol P450/min, respectively, for CYP1A2; and 25.9±6.6 µM and 134.3±12.4 nmol/nmol P450/min, respectively, for CYP2C19. Other human CYP isoforms including CYP2A6, CYP2C9, CYP2D6, CYP2E1 and CYP3A4 showed minimal or no effect on MH metabolism. The results suggested that MH was simultaneously a substrate and an inhibitor of CYP1A2 and CYP2C9, and MH had the potential to perpetrate drug-drug interactions with other CYP1A2 and CYP2C19 substrates.

## Introduction

Depression is a severe and recurrent mental disorder that often leads to a significant impairment of daily functions [1]–[3]. Antidepressants such as selective serotonin reuptake inhibitors (SSRIs) were commonly used to treat depression [4], [5]. However, adverse drug reactions (ADRs) to these drugs were difficult to predict and frequently interfered with the SSRI treatment, leading to treatment failure [6]–[8]. Antidepressants were facing weak prospects, as several pharmaceutical companies had been forced to withdraw their investment on the research of new antidepressants because of the noncompliance and discontinuation of the present SSRIs owing to ADRs [9], [10].

Common antidepressants were discontinued because they inhibited gastrointestinal kinetics [11], [12]. In contrast, meranzin hydrate (MH), an absorbed bioactive compound originating from the traditional Chinese medicine (TCM) Chaihu-Shugan-San (CSS) [13], [14], was considered a prospective candidate to treat depression because of its gastrointestinal prokinetic properties [15]–[17]. Xie *et al* reported that MH could increase the amplitude of contractility in the longitudinal and circular jejunum muscles in a dose-dependent manner [15], [16]. Furthermore, Huang *et al* reported that MH significantly accelerated the gastric emptying and intestinal transit in rats [17]. Therefore, MH was considered to be a prospective antidepressant better than other antidepressants.

Although MH showed anti-depression and gastrointestinal prokinetic effects in animal experiments, several questions were unanswered. In addition to the therapeutic effect, the safety of MH was one of the primary concerns. According to various reports, drug interactions could lead to serious adverse events or decreased drug efficacy. These interactions might occur through the inhibition or induction of hepatic and intestinal drug-metabolizing enzymes (e.g., CYPs) and transporters (e.g., p-glycoprotein) [18], [19]. CYP-mediated drug interactions was a major concern because CYP enzymes are involved in the phase metabolism of more than 70% of prescription drugs [20], and to the best of our knowledge, there was little published information about the metabolism of MH. In this study, we examined the CYP enzymes responsible for the metabolism of MH and the potential interactions of MH with typical substrates of the CYP enzymes *in vitro*.

## Methods

### Enzymes and chemicals

Recombinant human CYP enzymes (Bactosomes), pooled human liver microsomes (HLMs) from ten individual donors (Bactosomes) and NADPH were purchased from Cypex Ltd. (U.K.) and stored at −80°C until use. Meranzin hydrate (C_15_H_18_O_5_, MW: 278.30, assay≥98%) was obtained from Sigma-Aldrich (Shanghai, China). Furafylline, trans-2-phenylcyclopropylamine hydrochloride, ketoconazole, sulfaphenazole, quinidine, chlormethiazole hydrochloride and ticlopidine hydrochloride were purchased from the National Institutes for Food and Drug Control (Beijing, China). Phenacetin, coumarin, midazolam, tolbutamide, S-Mephenytoin, metoprolol, chlorzoxazone and the standards for their metabolites, including acetaminophen, 7-hydroxyl coumarin, 1-hydroxyl midazolam, 4-hydroxyl tolbutamide, 4-hydroxyl mephenytoin, α-hydroxyl metoprolol, 6-hydroxyl chlorzoxazone and irbesartan (the internal standard) were purchased from Sigma-Aldrich (Shanghai, China). All other chemicals and solvents were of high-performance liquid chromatography (HPLC) grade.

### Apparatus and operation conditions

The concentrations of the CYP substrates and their metabolites were quantified using a Waters 2695 separation module HPLC system (Waters Corp., Milford, Massachusetts, USA) coupled to a Quattro micro API triple quadrupole tandem mass spectrometer (Waters Corp., Milford, Massachusetts, USA) with an electrospray ionization source. The samples were separated on a HyPURITY C_18_ column (150 mm×2.1 mm, 5 µm, Thermo, USA) with a C_18_ security guard column (4.0 mm×3.0 mm, ID 5 µm). The mobile phases consisted of 20 mM ammonium formate and acetonitrile at a ratio of 60∶40. Aliquots of 20 µL were injected at a mobile phase flow rate of 0.3 mL/min. Multiple reaction monitoring was performed in the positive mode. The transitions are listed in table 1. The mass spectra of the metabolites formed in the incubations were identical to those of the corresponding authentic standards.

**Table 1:** Transitions and collision energies used in LC-MS/MS for the detection of meranzin hydrate, the probe substrates, metabolites and the internal standard.

Compound name	Precursor ion (m/z)	Product ion (m/z)	Collision energy (*eV*)
Meranzin hydrate	279.1	188.9	15
Acetaminophen	152	110	15
7-hydroxyl coumarin	162.9	107.0	20
1-hydroxyl midazolam	342	324	20
4-hydroxyl tolbutamide	287.0	171.0	15
4-hydroxyl mephenytoin	235.0	150.0	10
α-hydroxyl metoprolol	284.3	116.0	20
6-hydroxyl chlorzoxazone	195	138	20
Irbesartan (IS)	429.0	206.9	22

### General incubation conditions

The CYP isoform-specific probe reactions used were phenacetin O-deethylation (for CYP1A2), coumarin 7-hydroxylation (for CYP2A6), tolbutamide 4-hydroxylation (for CYP2C9), metoprolol α-hydroxylation (for CYP2D6), chlorzoxazone 6-hydroxylation (for CYP2E1), S-Mephenytoin 4-hydroxylation (for CYP2C19) and midazolam 1-hydroxylation (for CYP3A4). The kinetic study of MH was studied with HLMs, an incubation mixture that consisted of MH (as a substrate), the HLMs (0.5 mg/mL) or CYP isoforms (10 pmol) and 0.1 M sodium phosphate buffer (pH 7.4) in a total volume of 0.2 mL was pre-warmed for 5 min at 37°C without (control). The inhibitory effects of MH on the activities of seven different CYP isoforms were studied with the HLMs (and the expressed CYPs, when required), multiple concentrations of MH (as an inhibitor) were included in the above incubation mixture system. The reaction was initiated by the addition of 1 mg/mL triphosphopyridine nucleotide (NADPH). The final incubations were performed in a shaking water bath (37°C) for 30 min. The incubations were performed in triplicates, and the incubation conditions specific to each CYP isoform were within the linear range for the velocity of the reaction (the incubation time as well as the substrate and protein concentrations). All the reagents were dissolved in methanol, and the final solvent concentration in all incubations (including controls) was 1%. The reactions were stopped by adding 0.2 mL ice-cold acetonitrile containing irbesartan (114.9 ng/mL) as the internal standard. The samples were vortexed for 5 min. After centrifugation (12000×g for 10 min), the supernatants were transferred and aliquots of 20 µL were injected into the HPLC-MS/MS system for analysis.

### Kinetic analysis of MH

Kinetic analysis was performed for MH, and the data generated were used as a guide for selecting the appropriate concentrations of MH in the subsequent inhibition experiments. Thus, the kinetic parameters for the metabolism of MH was determined by incubating increasing concentrations of MH (0.5–100 µM) (without the inhibitor) at 37°C with the HLMs and NADPH under the incubation conditions. The equation of MH reaction velocity (*V*) in the HLMs or CYP isoforms was expressed as *V = (C_0_–C_t_)/*T*/C_p_*, where *C_0_* and *C_t_* represented the initial and final concentrations of MH in the incubation solution, respectively. T was the incubation time (min) and *Cp* was the protein concentration (mg/mL or nmol). All values were expressed as the mean±standard deviation (SD). The mean intrinsic clearance rate (*CL_int_*) for the *in vitro* incubation was estimated using *V_max_/K_m_*.

### Specific CYP isoforms screened for the metabolism of MH

To screen the specific CYP isoform responsible for the MH metabolism, we determined the inhibitory effect of specific inhibitors on the metabolism of MH in the HLMs. Inhibitors including furafylline (FUR, inhibitor for CYP1A2), trans-2-phenylcyclopropylamine (TRA, for CYP2A6, 1 µM), sulfaphenazole (SUL, for CYP2C9, 1 µM), quinidine (QUI, for CYP2D6, 1 µM), chlormethiazole (CHL, for CYP2E1, 5 µM), ticlopidine (TIC, for CYP2C19, 1 µM) and ketoconazole (KET, for CYP3A4, 1 µM) were separately incubated with MH (10 µM), the HLMs and NADPH under the same incubation conditions as mentioned above. The concentrations of the inhibitors used were approximately at their respective *IC_50_* values from previous reports [21]. The inhibitory effects of the above specific inhibitors on the metabolic clearance rate of MH were evaluated separately to screen the CYP isoforms responsible for the MH metabolism. The relative activity of the CYP isoforms was calculated by dividing the peak area of MH when incubated with the inhibitor with that of MH from the negative controls.

### Inhibition studies for *IC_50_* determination

A pilot inhibitory analysis of each CYP isoform was performed to determine the potency of inhibition and to select CYP isoforms for further detailed study of their inhibitions. MH (various concentrations of 0.5–100 µM) and a single CYP isoform-specific substrate (concentration at about the respective *K_m_* value) were used to determine the inhibitory effect of MH on specific CYP isoforms. Substrates including phenacetin, coumarin, tolbutamide, metoprolol, chlorzoxazone, S-Mephenytoin and midazolam were employed at concentrations of 10, 5, 100, 7.5, 40, 100 and 5 µM, respectively [21]. All incubation conditions were the same as mentioned above. The inhibitory effects on the CYP isoforms were investigated individually by incubating the HLMs in the absence or presence of MH. Incubation solution with the solvent that was used to dissolve MH was regarded as the negative control, whereas solutions containing the specific inhibitors mentioned above were regarded as the positive controls. The *IC_50_* values of MH were determined and compared with those of the specific inhibitors mentioned above (see Table 2).

**Table 2:** *IC_50_* and *K_i_* values of MH against human CYP isoforms compared with that of specific inhibitors reported in literature.

CYP	Activity	*IC_50_* (µM)		*K_i_* (µM)	
		MH	Specific inhibitor/reported values^a^	MH	Specific inhibitor/reported values^a^
CYP1A2	Phenacetin O-deethylation	4.47(3.10,6.23)^b^	FUR/1.4 [27]	4.56	FUR/3 [28]
CYP2A6	coumarin 7-hydroxylation	>100	TRA/0.42±0.07 [29]	-	TRA/0.17 [29]
CYP2C9	tolbutamide 4-hydroxylation	>100	SUL/0.3–1.5 [27], [30]	-	SUL/0.3 [28]
CYP2D6	metoprolol α-hydroxylation	>100	QUI/0.02–0.68 [27], [30]	-	SUL/0.027–0.4 [28], [31], [32]
CYP2E1	chlorzoxazone 6-hydroxylation	>100	DIE/21.30 [27]	-	CHL/12 [33]
CYP2C19	S-Mephenytoin 4-hydroxylation	10.91(8.84,13.45)^b^	TCL/0.52–1.6 [30]	42.65	TCL/1.2±0.5 [28]
CYP3A4	midazolam 1-hydroxylation	>100	KET/0.08–0.24 [28]	–	KET/0.015 [28]

a *IC_50_* and *K_i_* values of specific inhibitors were referred to the reported literatures. ^b^ represents 95% confidence interval. “–”represents the data that is not calculated. FUR, furafylline; TRA, trans-2-phenylcyclopropylamine hydrochloride; SUL, sulfaphenazole; QUI, quinidine; CHL, chlormethiazole hydrochloride; TIC, ticlopidine hydrochloride; KET, ketoconazole; DIE: diethyldithiocarbamate.

### Determination of *K_i_*

In pilot experiments (*IC_50_* determination), we noted that MH markedly inhibits CYP1A2 and CYP2C19, whereas its effect on the remaining CYPs (CYP2A6, CYP2C9, CYP2D6, CYP2E1 and CYP3A4) was minimal. Therefore, Dixon plots for the inhibition of CYP1A2 and CYP2C19 were determined by incubating the substrate probe at multiple concentrations with or without the test inhibitor at multiple concentrations with the HLMs and cofactors. The inhibition data obtained from the pilot experiments were used as a guide to generate appropriate probe substrate and test inhibitor concentrations for the determination of the *K_i_* values for each CYP isoform. The isoform-specific probe substrate concentrations used were 3 to 100 µM phenacetin for CYP1A2 and 15 to 120 µM S-mephenytoin for CYP2C19. The MH concentrations used were 0 to 100 µM.

### Calculations of enzyme kinetics and statistical method

To determine the major enzymes responsible for MH metabolism in HLMs, the metabolic clearance rate of the incubation solution without any specific inhibitor for MH was considered as 100%. The effects of the specific inhibitors on the metabolic clearance rate of MH were evaluated with SPSS one-way analysis of variance (SPSS Inc., Chicago, IL, USA). P<0.05 denoted significance in all cases. The apparent kinetic parameters of MH (*K_m_* and *V_max_*) were determined by fitting the Michaelis-Menten equation using the program GraphPad Prism Enzyme Kinetic 5 Demo (GraphPad Co. Ltd, San Diego, CA, USA). The equation was expressed as *V = V_max_[S]/(K_m_+[S])*. *K_m_* is the substrate concentration at which the reaction velocity is 50% of *V_max_*. To determine the inhibition of CYP isoforms, the activity of each CYP isoform was calculated using the metabolic clearance rate of its corresponding probe substrate. The metabolic clearance rate of the probe substrate was considered to be 100% when no specific inhibitor and MH were added in the incubation assay. The *IC_50_s* were determined by analyzing the plot of the logarithm of the inhibitor concentration versus the percentage of activity remaining after inhibition, using the SPSS software for Windows (version 11.5, SPSS, Chicago, IL). To calculate the *K_i_* values, the inhibition data were fit to different models of enzyme inhibition (competitive, noncompetitive, and uncompetitive) by nonlinear least-squares regression analysis using the GraphPad Prism 5 software (GraphPad Co. Ltd).

## Results

### Kinetic analysis of MH

**Figure 1:**
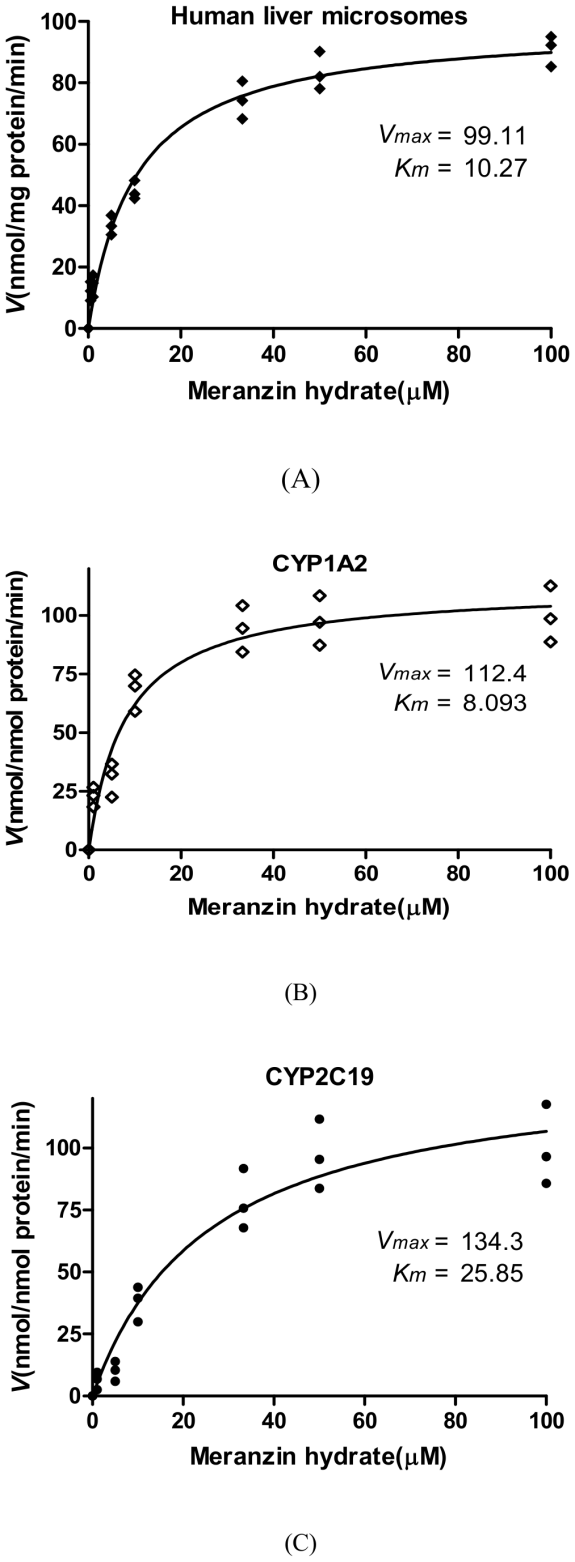
The concentration-velocity curve for meranzin hydrate metabolism after incubation with HLMs (A), recombinant CYP1A2 (B) and recombinant CYP2C19 (C). Note: The incubation conditions are described in the materials and methods section. The curve was automatically fitted using nonlinear regression and Michaelis-Menten equation, the data were obtained in triplicates.

Fig. 1. A, B and C show the metabolism of MH after incubation with the HLMs, CYP1A2 and CYP2C19. The kinetic plots indicated that the *K_m_* and *V_max_* values were 10.3±1.3 µM and 99.1±3.3 nmol/mg protein/min for the HLMs, 8.0±1.6 µM and 112.4±5.7 nmol/nmol P450/min for CYP1A2, 25.9±6.6 µM and 134.3±12.4 nmol/nmol P450/min for CYP2C19, respectively. The *in vitro CL_int_* values of MH in the HLMs, CYP1A2 and CYP2C19 were 9.7 mL/mg protein^−1^·min^−1^, 14.1 mL/nmol P450^−1^·min^−1^ and 5.2 mL/nmol P450^−1^·min^−1^, respectively.

### Specific CYP isoforms for the metabolism of MH

**Figure 2:**
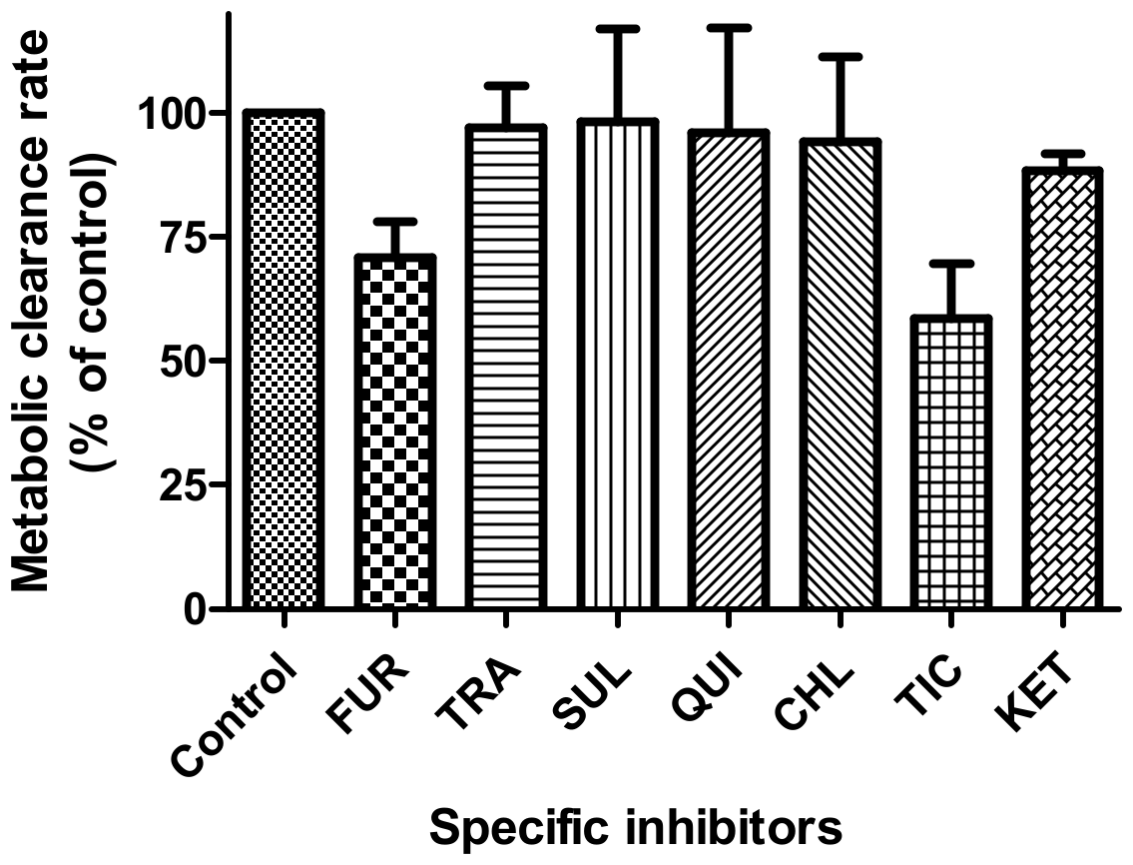
Effect of specific inhibitors on CYP-mediated MH (10 µM) metabolism in HLMs. Note: The concentrations of CHL was 5 µM and that for the other specific inhibitors was 1 µM. The incubation conditions are described in the materials and methods section. Each data point represents the average of triple determinations and error bars (n = 3). FUR, furafylline, the specific inhibitor for CYP1A2; TRA, trans-2-phenylcyclopropylamine, the specific inhibitor for CYP2A6; SUL, sulfaphenazole, the specific inhibitor for CYP2C9; QUI, quinidine, the specific inhibitor for CYP2D6; CLM, chlormethiazole, the specific inhibitor for CYP2E1; TIC, ticlopidine, the specific inhibitor for CYP2C19; and KET, ketoconazole, the specific inhibitor for CYP3A4. In the presence of FUR (1 µM) and TIC (1 µM), the metabolic clearance rate of MH decreased to 29.1% and 41.3% of that of the control, respectively, while the other inhibitors had no significant inhibitory effects on the metabolism of MH.

**Figure 3:**
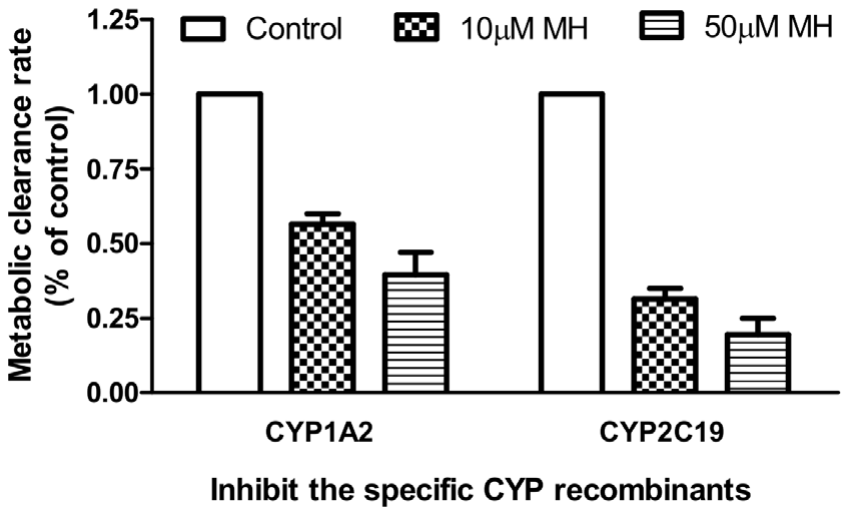
Effect on the metabolic clearance rate (MCR) of meranzin hydrate (MH) under the inhibition of recombinant CYP1A2 and CYP2C19. Note: 10 µM and 50 µM MH were incubated with the CYP recombinants and cofactors in the absence (control) or presence of the inhibitors by furafylline (1 µM) and ticlopidine (1 µM), respectively. Each point represents the average of triplicate incubations. The MCRs of MH were significantly decreased compared with that of the control for CYP1A2 and CYP2C19, for both concentrations of MH.

The inhibitory effects of the CYP specific inhibitors on the metabolic clearance rate of MH in the HLMs were shown in Fig. 2. The concentrations of FUR, TRA, SUL, QUI, TIC and KET were 1 µM, except for CHL, which was 5 µM. The concentrations were selected on the basis of previously reported *IC_50_* or *K_i_* values for the CYP isoforms to ensure adequate inhibitory selectivity, as well as maximal inhibitory potency. In the presence of FUR (1 µM) and TIC (1 µM), the metabolic clearance rate (MCR) of MH decreased to 29.1±7.2% and 41.3±11.1% of that of the control, respectively (Fig. 2). However, other inhibitors had no obvious inhibitory effects on the metabolism of MH. The screened enzymes were further confirmed by human recombinant CYPs by using the specific inhibitors, the MCRs of MH were decreased to 43.5% (MH, 10 µM) and 60.5% (MH, 50 µM) of that of the control for CYP1A2 and to 68.5% (MH, 10 µM) and 80.5% (MH, 50 µM) of that of the control for CYP2C19 (Fig. 3). The results indicated that CYP1A2 and CYP2C19 were possibly the major enzymes responsible for the metabolism of MH *in vitro*.

### Estimation of *IC_50_s*

**Figure 4:**
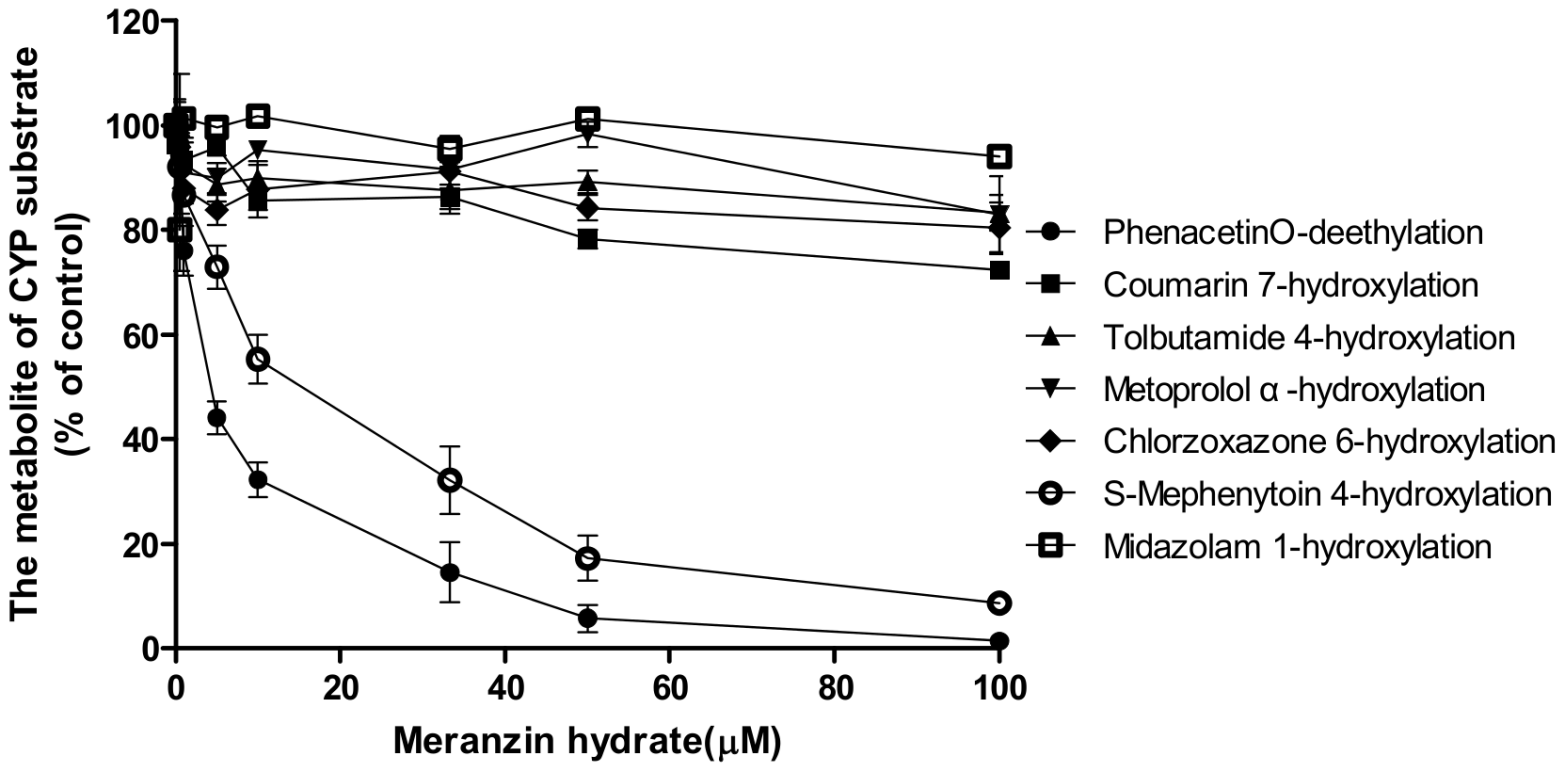
Effect of meranzin hydrate on the metabolic reactions of the seven CYP specific substrates in HLMs. Note: The incubation conditions are described in the materials and methods section. Each data point represents the average of triple determinations and error bars.

The inhibitory effects of multiple concentrations of MH (0.5–100 µM) on the activity of each CYP isoform determined by the metabolism of a single concentration of isoform-specific probe were tested with the HLMs (or expressed CYPs, when needed). MH showed potent inhibition of CYP1A2 (phenacetin O-deethylation) and CYP2C19 (S-mephenytoin 4-hydroxylation), with *IC_50s_* of 4.30 µM and 23.45 µM, respectively. The inhibitory effect of MH on the activity of CYP2A6, CYP2C9, CYP2D6, CYP2E1 and CYP3A4 was negligible (Fig. 4).

### Estimation of *K_i_* values

While the *IC_50_*s were qualitatively informative and help to address whether inhibition had occurred, their values were of limited use because they could be influenced by the substrate concentration selected, and it might not be accurate to use these parameters for the quantitative prediction of drug interactions *in vivo*. Therefore, we performed additional experiments designed to estimate the *K_i_* values. The preliminary inhibition data generated from a single probe substrate reaction were used to simulate the appropriate range of substrate and inhibitor concentrations for use in the construction of Dixon plots for the inhibition of the CYP isoforms by MH in the HLMs, from which precise *K_i_* values were estimated.

**Figure 5:**
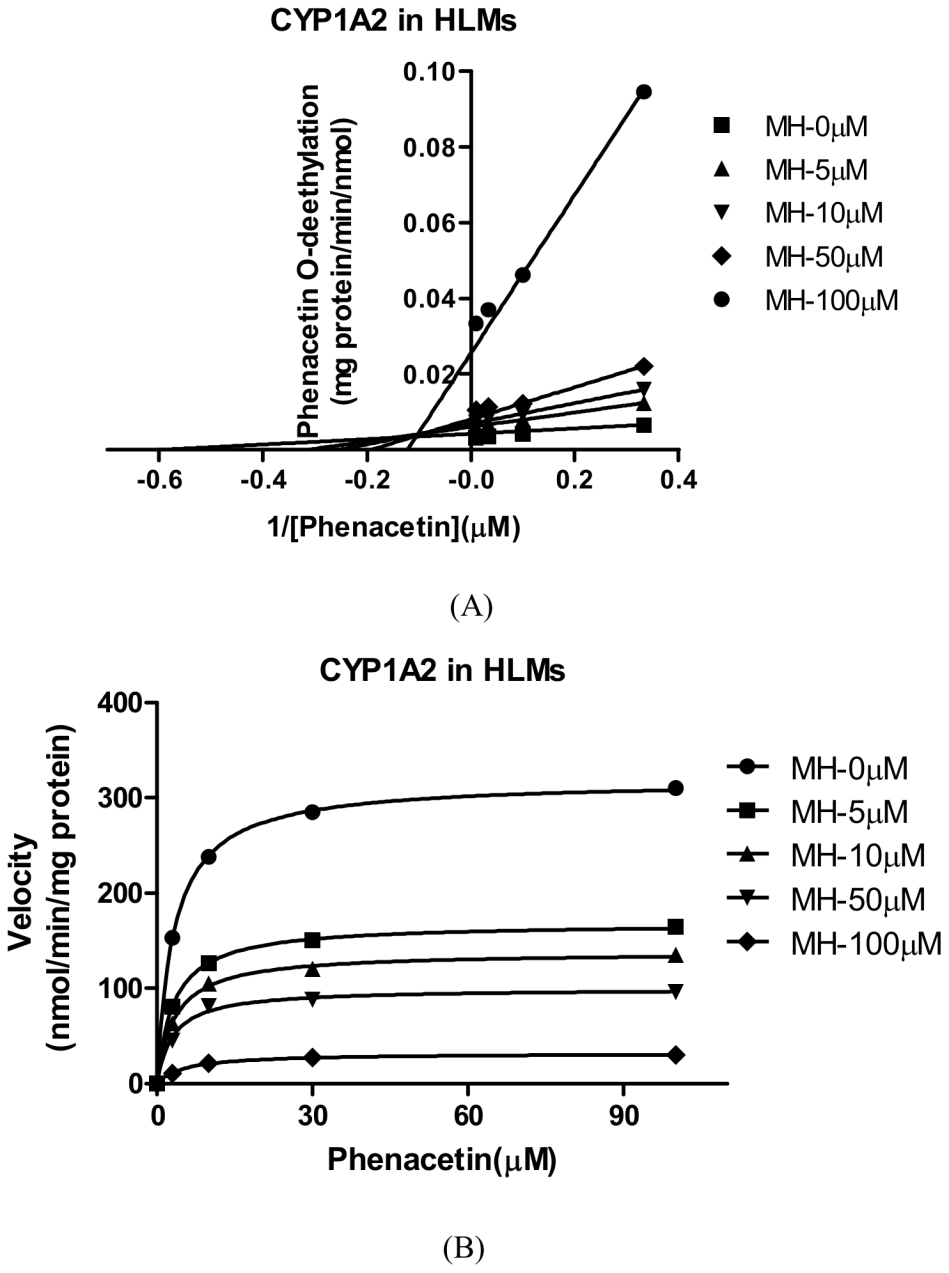
Double reciprocal (Lineweaver-Burk) plots for direct inhibition of phenacetin O-deethylation (A) and *K_i_* values. Note: The inhibition of CYP1A2 activity by MH can be best described as a mixed full inhibition mechanism by different concentrations of MH (0–100 µM) in HLM incubations (0.5 mg·mL^-1^ protein). The data points are mean values of triplicate incubations. Non-linear regression analysis of the phenacetin O-deethylation versus substrate concentration was performed to obtain *K_i_* values.

**Figure 6:**
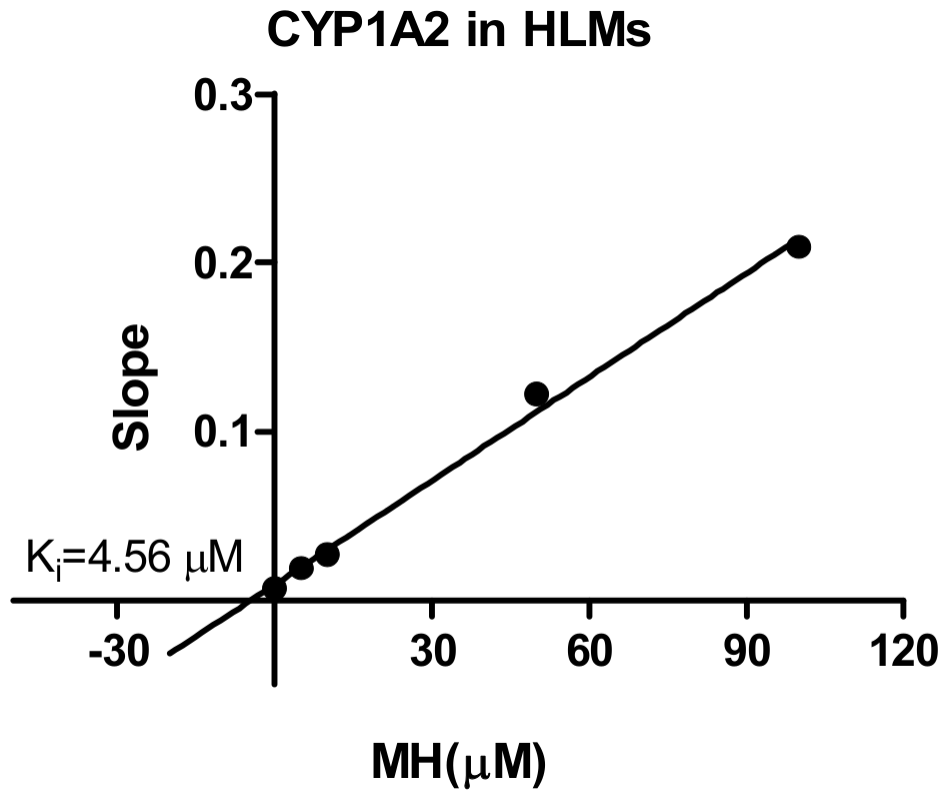
Secondary plot of CYP1A2 activity using the slopes of the primary Lineweaver-Burk plots versus concentrations of MH. Note: The effects of MH on the metabolism of phenacetin O-deethylation in human liver microsomes. Each point represents the mean of triplicate determinations.

**Figure 7:**
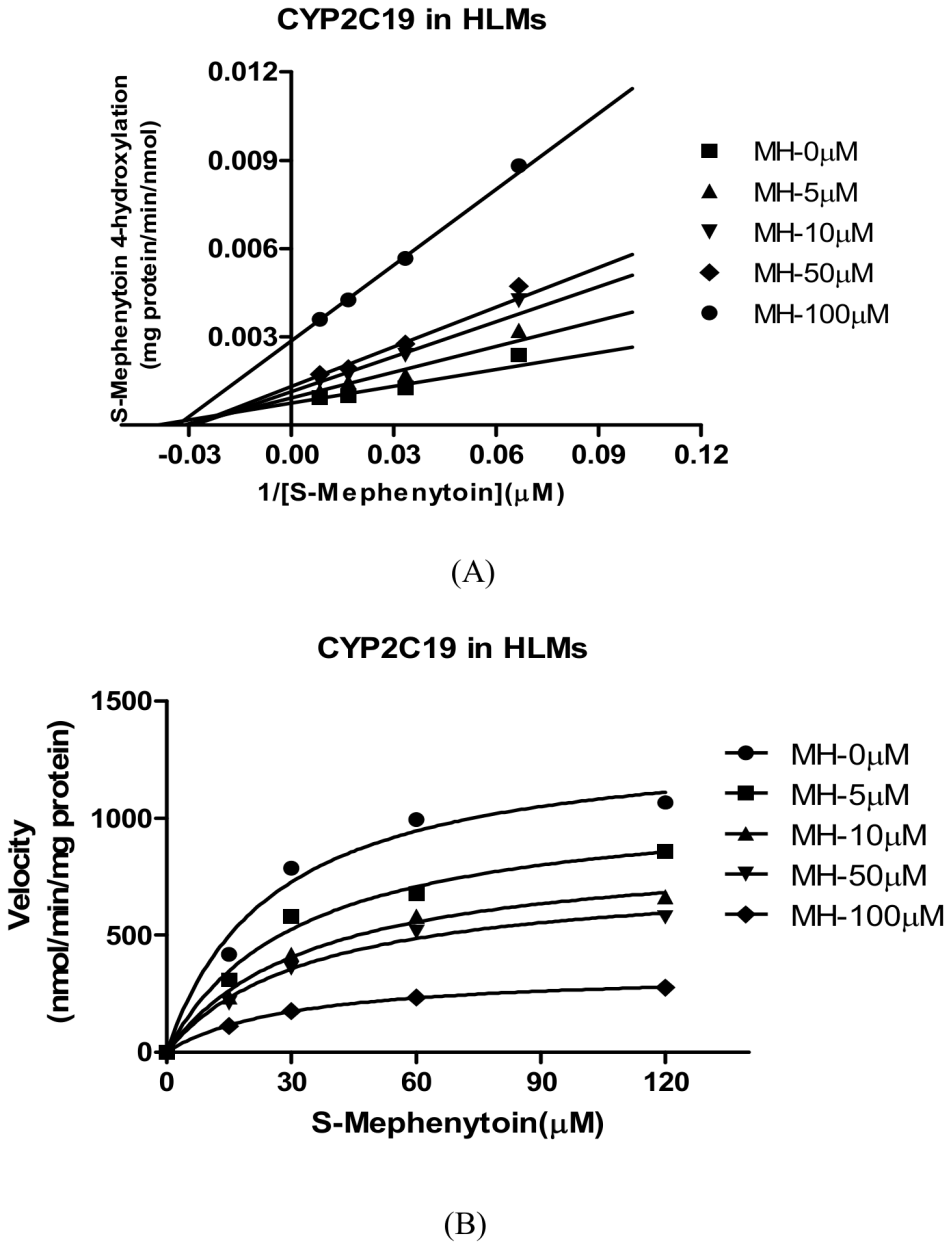
Double reciprocal (Lineweaver-Burk) plots for direct inhibition of S-Mephenytoin 4-hydroxylation (A) and *K_i_* values (B). Note: The inhibition of CYP1A2 activity by MH can be best described as a full competitive inhibition by different concentrations of MH (0–100 µM) in HLM incubations (0.5 mg·mL^-1^ protein). The data points are mean values of triplicate incubations. Non-linear regression analysis of the S-Mephenytoin 4-hydroxylation versus substrate concentration was performed to obtain *K_i_* values.

**Figure 8:**
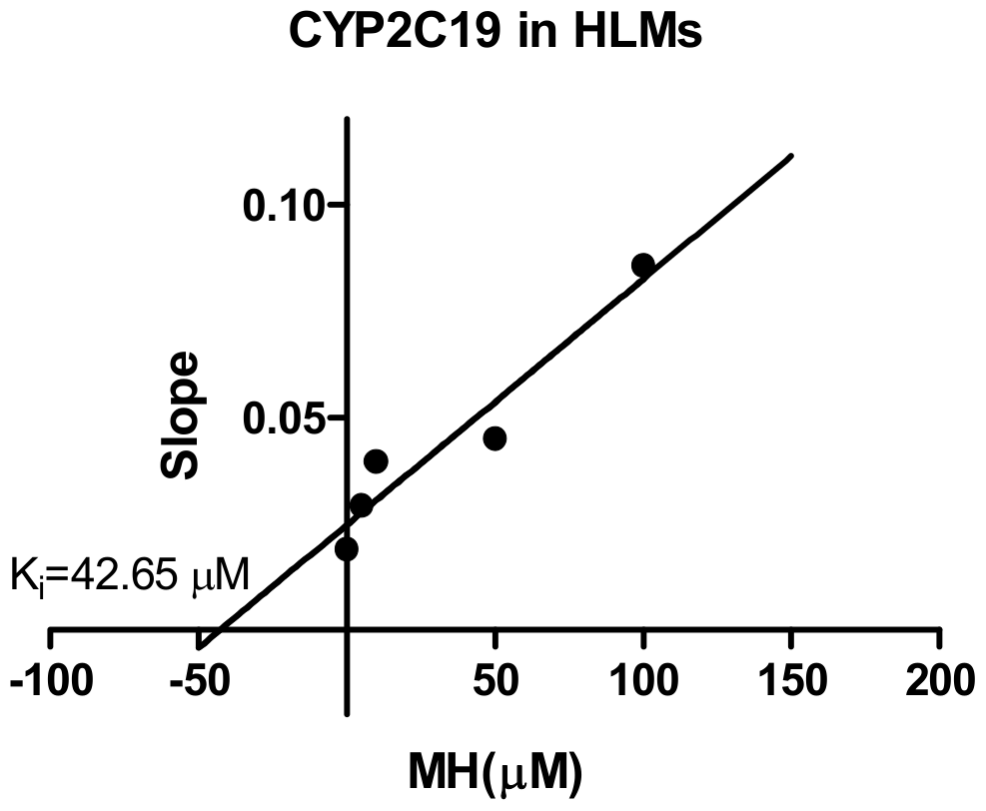
Secondary plot of CYP2C9 activity using the slopes of the primary Lineweaver-Burk plots versus concentrations of MH. Note: illustrating the effects of MH on the metabolism of S-Mephenytoin via 4-hydroxylation in human liver microsomes. Each point represents the mean of triplicate determinations.

For CYP1A2, the *K_i_* values were determined using phenacetin as the probe substrate. Of all CYPs tested, CYP1A2 was the most sensitive to the inhibition by MH (Table 2). The representative Dixon plots for the inhibition of CYP1A2 in the HLMs were shown in Fig. 5. Visual inspection of the Dixon plots and further analysis of the parameters of the enzyme inhibition models suggested that the inhibition data fit well to a competitive type of inhibition. The *K_i_* values estimated by using a nonlinear regression model for the competitive enzyme inhibition of CYP1A2-catalyzed phenacetin O-deethylation in the HLMs were less than 5 µM (Table 2, Fig. 6). Fig. 7. Shows the Dixon plots for the inhibition of CYP2C19 by MH in the HLMs. MH inhibited CYP2C19 competitively, with estimated *K_i_* values of 42.65 µM (Table 2, Fig. 8).

## Discussion

Depression is an incapacitating psychiatric ailment and widely recognized as one of the three major diseases of the new century, besides cancer and AIDS [22]. Antidepressants are commonly used to treat depression; however, most of them were withdrawn from treatment due to serious side effects [6]. Therefore, new drugs with high efficiency and low toxicity are urgently required for treating depression. MH, an absorbed bioactive compound from Chaihu-Shugan-San (CSS), displayed potent preclinical anti-depression and gastrointestinal prokinetic functions [13], [14], rendering it a prospective candidate to treat depression. However, several questions need to be answered in order for MH to be introduced as a new antidepressant.

The drug interactions of MH were one of the critical questions to be resolved. These interactions might occur through the inhibition or induction of drug-metabolizing enzymes and lead to serious adverse events or decreased drug efficacy [12]. The cytochrome P450 (CYP) enzymes are a large family of drug-metabolizing enzymes that play a critical role in Phase I drug metabolism, and most of the endogenous and exogenous substances are the substrates of CYPs [13]. CYP-mediated drug interactions is a major concern; the inhibition of a CYP enzyme can cause an increase in drug plasma levels via decreased drug metabolism, which could result in significant adverse reactions or toxicities. Therefore, inhibition-based drug interactions are a primary cause of clinically significant drug interactions [14]. The major CYPs involved in the hepatic metabolism of most drugs are CYP1A2, CYP2A6, CYP2C9, CYP2D6, CYP2E1, CYP2C19 and CYP3A4 [14]; therefore, screening the CYPs for the metabolism of MH could predict its drug interactions.

In the present study, we have shown that MH is a substrate of CYP1A2 and CYP2C19, especially CYP1A2. Our data and findings suggested that MH was unlikely to alter the pharmacokinetics of drugs metabolized by CYP2A6, CYP2C9, CYP2D6, CYP2E1 and CYP3A4. Of the seven CYPs studied, CYP1A2 and CYP2C19 were screened out as participating in the metabolism of MH, by using specific inhibitors on CYP in the HLMs. The MCRs of MH were decreased to 29.1% and 41.3% of that of the control, respectively (Fig. 2). The results were further confirmed by the recombinant CYP1A2 and CYP2C19 experiment; after inhibiting the activities of the enzymes, the MCRs of MH were significantly decreased to 43.5% (MH, 10 µM) and 60.5% (MH, 50 µM) of that of the control for CYP1A2 and to 68.5% (MH, 10 µM) and 80.5% (MH, 50 µM) of that of the control for CYP2C19 (Fig. 3). The kinetics study indicated that the *K_m_* and *V_max_* values of MH were 10.3±1.3 µM and 99.1±3.3 nmol/mg protein/min for the HLMs, 8.0±1.6 µM and 112.4±5.7 nmol/nmol P450/min for CYP1A2 and 25.9±6.6 µM and 134.3±12.4 nmol/nmol P450/min for CYP2C19, respectively (Fig. 1). CYP1A2 and CYP2C19 might be the major enzymes involved in the metabolism of MH *in vitro*.

Our study showed that CYP1A2 was most sensitive to the inhibition by MH (*IC_50_* = 4.47 µM), and CYP2C19 was less sensitive to the inhibition by MH (*IC_50_* = 10.91 µM). CYP2A6, CYP2C9, CYP2D6, CYP2E1 and CYP3A4 were hardly sensitive to the inhibition of MH (*IC_50s_* were all more than 100 µM) (Table 2). The *IC_50_* value of CYP1A2 for the O-deethylation of phenacetin was 3.2-fold higher than that of FUR in the HLMs. The effect of MH on the O-deethylation of phenacetin metabolism was further assayed using CYP1A2 (10 pmol), by the co-incubation of phenacetin (final concentration of 3, 10, 30 or 100 µM) with MH (final concentrations of 0, 5, 10, 50 and 100 µM) for 30 min. The *K_i_* values were evaluated using the Dixon plot method (Fig. 5). The results showed that MH, at concentrations lower than 100 µM, significantly inhibited the CYP1A2-catalysed O-deethylation of phenacetin with *K_i_* = 4.56 µM, which was approximately 1.5-fold higher than that of FUR in the HLMs. The type of competition appears to be competitive inhibition. Fig. 7. Showed the Dixon plots for the inhibition of CYP2C19 by MH in the HLMs. The *IC_50_* value of CYP2C19 for 4-hydroxyl of S-Mephenytoin was 6.8–21.8-fold higher than that of TCL in the HLMs. MH was a moderate inhibitor for CYP2C19, with an estimated *K_i_* value of 42.65 µM (Table 2). According to Kong *et al* [23], the potency of a test compound could be classified according to its *IC_50_* values, as potent, if *IC_50_*≤20 µg/mL or ≤10 µM; moderate, if *IC_50_* 20–100 µg/mL or 10–50 µM; or weak, if *IC_50_*≥100 µg/mL or ≥50 µM. Thus, MH is a potent inhibitor for CYP1A2, a moderate inhibitor for CYP2C19 and a weak inhibitor for the other five CYPs tested in this study.

Drug interactions associated with the induction or inhibition of CYP enzymes was among the most important causes of side effects in humans, and the inhibition effect was considered as the most common mechanism involved in CYP-associated drug-drug interactions. The results of the present work indicated that MH had the potential to interact with a wide range of xenobiotics or endogenous chemicals that were CYP1A2 or CYP2C19 substrates during clinical treatment. Because these two enzymes are involved in the clearance of 6–7% of currently marketed drugs, there is a strong possibility that MH would be co-prescribed with drugs that interact and thus elicit frequent and severe adverse drug interactions in the population. However, it is important to note that the extent of drug interactions with MH might highly vary among individuals due to genetic polymorphisms of CYP1A2 and CYP2C19. In previous studies, allelic variants in the genes encoding CYP1A2 and CYP2C19 had been shown to affect enzyme activities[24]–[26]. The alleles were classified as belonging to three major phenotypes: extensive metabolism, intermediate metabolism and poor metabolism. Thus, the effect of the gene polymorphisms of these two enzymes should be considered during clinical trials for MH in the future.

In conclusion, our results show that MH was simultaneously a substrate and an inhibitor of CYP1A2 and CYP2C9, and MH had the potential to perpetrate drug-drug interactions with other CYP1A2 and CYP2C19 substrates. Because CYP1A2 and CYP2C19 are polymorphic enzymes, the drug interactions between MH and the substrates of these enzymes should be considered during future experiments.

## References

[1] Tombal B (2010) Prostate cancer, depression, and risk of suicide: should we pay more attention? Eur Urol 396–397. PMID:19962228. 10.1016/j.eururo.2009.11.039 19962228

[2] Walker J, Hansen CH, Butcher I, Sharma N, Wall L, et al**.** (2011) Thoughts of death and suicide reported by cancer patients who endorsed the “suicidal thoughts” item of the PHQ-9 during routine screening for depression. Psychosomatics 52:: 424–427. PMID:21907060. 10.1016/j.psym.2011.02.003 21907060

[3] Plesnicar BK (2014) Efficacy and tolerability of agomelatine in the treatment of depression. Patient Prefer Adherence 8:: 603–612. PMID:24833894. 10.2147/PPA.S42789 PMC4014359 24833894

[4] De Crescenzo F, Perelli F, Armando M, Vicari S (2014) Selective serotonin reuptake inhibitors (SSRIs) for post-partum depression (PPD): a systematic review of randomized clinical trials. J. Affect Disord 152–154: 39–44. PMID:24139299. 10.1016/j.jad.2013.09.019 24139299

[5] Kocsis JH (2013) Review: SSRIs and TCAs equally effective at treating chronic depression and dysthemia; SSRIs are associated with fewer adverse events than TCAs. Evid Based Ment Health 16: 82. PMID:23604275. 10.1136/eb-2013-101268 PMC13421620 23604275

[6] Lee YC, Lin CH, Lin MS, Lin JW, Chang CH, et al**.** (2013) Effects of selective serotonin reuptake inhibitors versus tricyclic antidepressants on cerebrovascular events: a nationwide population-based cohort study. J Clin Psychopharmacol 33:: 782–789. PMID:24091857. 10.1097/JCP.0b013e31829c970e 24091857

[7] Weeke P, Jensen A, Folke F, Gislason GH, Olesen JB, et al**.** (2012) Antidepressant Use and Risk of Out-of-Hospital Cardiac Arrest: A Nationwide Case-Time-Control Study. Clinical Pharmacology & Therapeutics 92:: 72–79. PMID:22588605. 10.1038/clpt.2011.368 22588605

[8] Graf H, Walter M, Metzger CD, Abler B (2013) Antidepressant-related sexual dysfunction - Perspectives from neuroimaging. Pharmacol Biochem Behav. PMID:24333547. 10.1016/j.pbb.2013.12.003 24333547

[9] Chancellor D (2011) The depression market. Nat Rev Drug Discov 10:: 809–810. PMID:22037032. 10.1038/nrd3585 22037032

[10] McInerney M, Mellor JM, Nicholas LH (2013) Recession depression: mental health effects of the 2008 stock market crash. J Health Econ 32:: 1090–1104. PMID:24113241. 10.1016/j.jhealeco.2013.09.002 PMC3874451 24113241

[11] Schurink B, Tielemans MM, Aaldering BR, Eikendal T, Focks JJ, et al**.** (2014) Antidepressants and gastrointestinal symptoms in the general Dutch adult population. J Clin Psychopharmacol 34:: 66–71. PMID:24346754. 10.1097/JCP.0000000000000055 24346754

[12] Tan E, Smith CH, Goldman RD (2013) Antidepressants for functional gastrointestinal disorders in children. Can Fam Physician 59:: 263–264. PMID:23486795. PMC3596202 23486795

[13] Kim SH, Han J, Seog DH, Chung JY, Kim N, et al**.** (2005) Antidepressant effect of Chaihu-Shugan-San extract and its constituents in rat models of depression. Life Sci 76:: 1297–1306. PMID:15642599. 10.1016/j.lfs.2004.10.022 15642599

[14] Chen S, Asakawa T, Ding S, Liao L, Zhang L, et al**.** (2013) Chaihu-Shugan-San administration ameliorates perimenopausal anxiety and depression in rats. PLoS One 8:: e72428. PMID:24015243. 10.1371/journal.pone.0072428 PMC3754981 24015243

[15] Xie Y, Huang X, Hu SY, Zhang YJ, Wang Y, et al**.** (2013) The involvement of AMPA-ERK1/2-BDNF pathway in the mechanism of new antidepressant action of prokinetic meranzin hydrate. Amino Acids 44:: 413–422. PMID:22782214. 10.1007/s00726-012-1347-2 22782214

[16] Xie Y, Huang X, Hu SY, Qiu XJ, Zhang YJ, et al**.** (2013) Meranzin hydrate exhibits anti-depressive and prokinetic-like effects through regulation of the shared alpha2-adrenoceptor in the brain-gut axis of rats in the forced swimming test. Neuropharmacology 67:: 318–325. PMID:23063894. 10.1016/j.neuropharm.2012.10.003 23063894

[17] Huang W, Huang X, Xing Z, Qiu X, Wang Y, et al**.** (2011) Meranzin hydrate induces similar effect to Fructus Aurantii on intestinal motility through activation of H1 histamine receptors. J. Gastrointest Surg 15:: 87–96. PMID:21061180. 10.1007/s11605-010-1374-9 21061180

[18] Johnson TN, Kerbusch T, Jones B, Tucker GT, Rostami-Hodjegan A, et al**.** (2009) Assessing the efficiency of mixed effects modelling in quantifying metabolism based drug-drug interactions:using in vitro data as an aid to assess study power. Pharm Stat 8:: 186–202. PMID:19291743. 10.1002/pst.373 19291743

[19] Tang C, Lin JH, Lu AY (2005) Metabolism-based drug-drug interactions: what determines individual variability in cytochrome P450 induction? Drug Metab Dispos 33:: 603–613. PMID:15673596. 10.1124/dmd.104.003236 15673596

[20] Keers R, Aitchison KJ (2011) Pharmacogenetics of antidepressant response. Expert Rev Neurother 11:: 101–125. PMID:21158559. 10.1586/ern.10.186 21158559

[21] Xu C, Desta Z (2013) In vitro analysis and quantitative prediction of efavirenz inhibition of eight cytochrome P450 (CYP) enzymes: major effects on CYPs 2B6, 2C8, 2C9 and 2C19. Drug Metab Pharmacokinet 28:: 362–371. PMID:23385314. 10.2133/dmpk.dmpk-12-rg-124 PMC4075192 23385314

[22] Greenberg PE, Stiglin LE, Finkelstein SN, Berndt ER (1993) Depression: a neglected major illness. J Clin Psychiatry 54:: 419–424. PMID:8270584. 8270584

[23] Kong WM, Chik Z, Ramachandra M, Subramaniam U, Aziddin RE, et al**.** (2011) Evaluation of the effects of Mitragyna speciosa alkaloid extract on cytochrome P450 enzymes using a high throughput assay. Molecules 16:: 7344–7356. PMID:21876481. 10.3390/molecules16097344 PMC6264431 21876481

[24] Chen Y, Tu JH, He YJ, Zhang W, Wang G, et al**.** (2009) Effect of sodium tanshinone II A sulfonate on the activity of CYP1A2 in healthy volunteers. Xenobiotica; the fate of foreign compounds in biological systems. 39:: 508–513. PMID:19534587. 10.1080/00498250902951763 19534587

[25] Song L, Du Q, Jiang X and Wang L (2014) Effect of CYP1A2 polymorphism on the pharmacokinetics of agomelatine in Chinese healthy male volunteers. Journal of clinical pharmacy and therapeutics. 39:: 204–209. PMID:24372004. 10.1111/jcpt.12118 24372004

[26] Yamamoto Y, Takahashi Y, Imai K, Miyakawa K, Nishimura S, et al**.** (2013) Influence of CYP2C19 polymorphism and concomitant antiepileptic drugs on serum clobazam and N-desmethyl clobazam concentrations in patients with epilepsy. Therapeutic drug monitoring 35:: 305–312. PMID:23666564. 10.1097/FTD.0b013e318283b49a 23666564

[27] Li X, Wang K, Wei W, Liu YY, Gong L (2013) In vitro metabolism of brucine by human liver microsomes and its interactions with CYP substrates. Chem Biol Interact 204:: 140–143. PMID:23707193. 10.1016/j.cbi.2013.05.007 23707193

[28] Bourrie M, Meunier V, Berger Y, Fabre G (1996) Cytochrome P450 isoform inhibitors as a tool for the investigation of metabolic reactions catalyzed by human liver microsomes. J Pharmacol Exp Ther 277:: 321–332. PMID:8613937. 8613937

[29] Taavitsainen P, Juvonen R, Pelkonen O (2001) In vitro inhibition of cytochrome P450 enzymes in human liver microsomes by a potent CYP2A6 inhibitor, trans-2-phenylcyclopropylamine (tranylcypromine), and its nonamine analog, cyclopropylbenzene. Drug Metab Dispos 29:: 217–222. PMID:11181487. 11181487

[30] Qin CZ, Ren X, Tan ZR, Chen Y, Yin JY, et al**.** (2014) A high-throughput inhibition screening of major human cytochrome P450 enzymes using an in vitro cocktail and liquid chromatography-tandem mass spectrometry. Biomed Chromatogr 28:: 197–203. PMID:23946123. 10.1002/bmc.3003 23946123

[31] Ching MS, Blake CL, Ghabrial H, Ellis SW, Lennard MS, et al**.** (1995) Potent inhibition of yeast-expressed CYP2D6 by dihydroquinidine, quinidine, and its metabolites. Biochem Pharmacol 50:: 833–837. PMID:7575645. 10.1016/0006-2952(95)00207-g 7575645

[32] Ring BJ, Binkley SN, Vandenbranden M, Wrighton SA (1996) In vitro interaction of the antipsychotic agent olanzapine with human cytochromes P450 CYP2C9, CYP2C19, CYP2D6 and CYP3A. Br J Clin Pharmacol 41:: 181–186. PMID:8866916. 10.1111/j.1365-2125.1996.tb00180.x 8866916

[33] Gebhardt AC, Lucas D, Menez JF, Seitz HK (1997) Chlormethiazole inhibition of cytochrome P450 2E1 as assessed by chlorzoxazone hydroxylation in humans. Hepatology 26:: 957–961. PMID:9328319. 10.1002/hep.510260423 9328319

